# Legal Regulation of Sodium Consumption to Reduce Chronic Conditions

**DOI:** 10.5888/pcd13.150545

**Published:** 2016-02-18

**Authors:** James G. Hodge, Leila F. Barraza

**Affiliations:** Author Affiliations: Leila F. Barraza, University of Arizona, Tucson, Arizona.

## Abstract

In the United States, tens of thousands of Americans die each year of heart disease, stroke, or other chronic conditions tied to hypertension from long-term overconsumption of sodium compounds. Major strides to lower dietary sodium have been made over decades, but the goal of reducing Americans’ daily consumption is elusive. The Food and Drug Administration (FDA) has been urged to consider stronger regulatory limits on sodium, especially in processed and prepared foods. Still, FDA categorizes salt (and many other sodium compounds) as “generally recognized as safe,” meaning they can be added to foods when ingested in reasonable amounts. Legal reforms or actions at each level of government offer traditional and new routes to improving chronic disease outcomes. However, using law as a public health tool must be assessed carefully, given potential trade-offs and unproven efficacy.

## Introduction

Sodium is essential to human life, but its overconsumption (primarily through salt) carries substantial public health complications. An estimated 1.65 million deaths from cardiovascular disease globally in 2010 were attributed to excessive sodium intake over time (1). Domestically, tens of thousands of Americans perish each year from heart disease, stroke, or other chronic conditions tied to hypertension (high blood pressure) from long-term overconsumption of sodium compounds (2). Nearly 70 years since its negative public health impact was initially observed in 1948 (3), overconsumption of sodium has become a public health crisis in the United States.

Major strides to lower dietary sodium have been made or attempted over decades (as discussed below), but the goal of reducing Americans’ daily consumption is elusive. National calls for broader public health responses have been made repeatedly by academics, doctors, and others (3). The Food and Drug Administration (FDA) has been urged to consider stronger regulatory limits on sodium, especially in processed and prepared foods. To date, FDA still categorizes salt (and many other sodium compounds) as “generally recognized as safe” (GRAS) (4) when ingested in reasonable amounts (3).

Public health and medical officials do not all agree on how much is too much salt. The American Heart Association recommends that daily sodium consumption should not exceed 1,500 mg (5). Federal dietary guidelines suggest limiting sodium intake to no more than 2,300 mg for individuals aged 14 or older and 1,500 mg for individuals with hypertension or prehypertension (6). An Institute of Medicine panel proposes similarly a Tolerable Upper Intake Limit of 2,300 mg for persons 14 years or older and average daily intake level of 1,500 mg for persons aged 9 to 51 years (7).

Under any of these standards, Americans’ actual sodium consumption is excessive. More than 90% of American children and close to 90% of adults ingest salt in excess of the Dietary Guidelines for Americans recommendations (8). Gradual overconsumption is linked to increases in blood pressure because salt can cause the body to retain extra fluids that burden the heart (9). Nearly 30% of American adults may have hypertension (10), placing them at substantial risk for cardiovascular diseases (which collectively contributed to approximately one-third of US deaths in 2011) (7).

Against this backdrop, we consider actual or potential legal options based on our research and policy assessments to lower the abundance of salt in the food supply substantially. Legal reforms or actions at each level of government offer traditional and new routes to improving chronic disease outcomes. However, using law as a public health tool must be assessed carefully, given unsettled science over the amount of sodium that produces negative health effects, potential legal and policy trade-offs, and unproven costs and efficacy of varied legal interventions.

## Law and Policy Options to Limit Sodium Consumption

Despite substantial public health repercussions of excessive consumption, regulating sodium intake has historically relied on voluntary efforts designed to encourage consumers to choose healthier foods and food manufacturers, retailers, and servers to provide more healthful options. As a result, consumers are better educated about the risks of sodium and how it is added to foods for flavor, for texture, or as a preservative. Processed food manufacturers and retailers have voluntarily lowered sodium amounts in their products. Schools, restaurants, theaters, and senior-living establishments have reduced sodium in prepared foods through innovative public–private partnerships (11). These and many other efforts show promise in addressing communal health concerns (7), but their impact is piecemeal at best. Average sodium consumption among Americans actually increased 55% from the mid-1980s to 2000 (12), correlated with a cascade of largely preventable health conditions. Across the United States, public health officials and policy makers are re-examining approaches to limiting the sodium consumption of various populations. These approaches include an increasing array of legal or policy options that entail governmental interventions in the interest of protecting the public’s health.

### Menu and label warnings

Most packaged foods have posted sodium content (generally accurate within 20% of the actual amount as per federal regulation) on their labels since 1986 (7), but similar “on the spot” information about prepared foods is largely absent. FDA’s forthcoming menu labeling requirements for restaurants and certain vendors focus largely on caloric posting and not so much on sodium (although such information is available on request) (13). However, FDA’s new regulations do not preempt state or local laws requiring menu postings of food warnings, most likely including sodium content. Multiple jurisdictions (eg, California, Oregon, Philadelphia, Seattle-King County) have already implemented menu labeling laws aimed at substantially curbing sodium ingestion. In September 2015, New York City’s Board of Health expanded its efforts as part of the 2009 National Salt Reduction Initiative by passing a regulation requiring chain restaurants (with 15 or more locations) to feature sodium warning labels (depicting the image of a saltshaker surrounded by a black triangle) on any menu item containing more than 2,300 mg (14).

New York City’s regulations were set to take effect on December 1, 2015, but the National Restaurant Association sued on December 3 challenging the expansion of the city’s initiative on constitutional and other grounds (15). Among its claims, the association alleges violations of separation of powers, substantive due process, and freedom of speech principles (to the extent that restauranteurs must post scientifically controversial information about salt content). It also claims that the initiative is preempted by the federal National Labeling and Education Act and generally unauthorized by FDA. Initial hearings have been scheduled, but it will be months before case results are known.

### Focus on minors

Virtually everyone agrees that reducing sodium consumption by minors can improve their life-time health substantially. In 2012, a prominent national theme park operator announced it would reduce sodium levels in children’s meals at its parks by 25% and limit advertising of high sodium products on its media outlets (16). Industry-led voluntary efforts like these are beneficial, but more substantial legal interventions concentrate on cutting sodium in minors’ diets at school and in residential childcare institutions. The federal Healthy, Hunger-Free Kids Act of 2010 required numerous changes in sodium levels in children’s school lunches and snack foods (17). Schools must reduce the sodium content of meals offered through the National School Lunch Program and the School Breakfast Program by 25% to 50% (based on the Institute of Medicine recommendations [7]) by July 1, 2022. New standards also limit snacks sold in school stores, vending machines, or à la carte to 230 mg per item in 2014 and 200 mg by July 1, 2016 (18).

### Litigation

In the fight to lower sodium consumption, litigation may be used to 1) compel greater governmental regulation, 2) require restaurants and other food servers to disclose sodium levels publicly, and 3) seek truth in advertising (discussed below). In 2005, the Center for Science in the Public Interest (CSPI) petitioned FDA to reconsider the GRAS classification of sodium, set ceilings on the amount of sodium in processed foods, require health warnings on packaged salt, and reduce the recommended daily intake for sodium (19). CSPI later sued FDA in October 2015, alleging that FDA failed to grant or deny its petition despite holding a hearing on the matter (20).

In 2010, one consumer brought a class action suit in Illinois against a national restaurant chain for its failure to disclose excessive sodium content in certain menu items (21). One of the restaurant’s breakfast entrees, for example, purportedly included 5,690 mg of sodium. It was alleged that the company knowingly concealed from consumers information about the excessive amount of sodium in this and other meals. Although the district court dismissed the case for failure to state a claim, extensive media coverage may have contributed to consumer awareness of excessive sodium content in restaurant foods.

### Truth in commercial advertising

Greater public health education of sodium’s dangers coupled with pledges from food manufacturers to gradually decrease sodium content in their products have resulted in more “low sodium” products. The Centers for Disease Control and Prevention specifically encourages Americans to use such products pursuant to the federal Dietary Approaches to Stop Hypertension (DASH) initiative (22). In response, manufacturers created products labeled and advertised as “reduced sodium” or “low sodium.” Such claims can be made consistent with First Amendment commercial speech principles as long as their statements are not false or misleading.

Assessing the veracity of manufacturers’ commercial messages, however, can be dicey given disagreements over the proper benchmarks denoting appropriate or safe limits of sodium (7). Competing terminology can confuse consumers. Labeling canned products as “low sodium,” “reduced sodium,” or “light sodium” may mean about the same thing to purchasers even though each message denotes appreciably different amounts of sodium via federal guidance. Patently false claims are legally actionable. In 2010, a national soup manufacturer was sued in New Jersey by a class of consumers for allegedly misrepresenting the salt content in its products. Consumers claimed that the company’s “25% Less Sodium” tomato soup contained the same amount of sodium (480 mg) per serving as its regular tomato soup (23). The company settled the case for $1.05 million and agreed to change its labels. Later, in 2013, the same company and the American Heart Association were sued over their dual allowance of “heart healthy” claims for soups with exceptionally high sodium content.

### Tax, spend, and ban

Three public health legal tools often used to deal with products associated with negative public health impacts include 1) taxes (to decrease consumer use and raise public health revenues), 2) spending incentives (to directly influence industry or consumer choices through financial rewards), and 3) bans (to remove a product from the market or limit specific consumers’ access) (24). These lawful interventions are used extensively to regulate potentially harmful consumables such as tobacco, illicit drugs, alcohol, and even caffeine. On first glance, these powers seem to lack utility regarding sodium — and perhaps for good reason. Unlike other “vice” products, sodium is inexpensive and pervasive in nearly every category of foods and beverages. In small amounts, salt is not only safe but essential. Salt makes food taste better and last longer with no added calories. Taxing or banning salt or other sodium products may seem unjustified, especially considering Americans’ common political objections to “nanny state” interventions.

Yet, in reality, each of these regulatory tools applies to sodium already. Salt as an ingredient is not taxed directly via state or local governments, but salty snacks and other foods are. In 2015, the Navajo Nation imposed a 2% tax on all junk food sold on its reservation, including an array of high sodium products of “minimal-to-no nutritional value.” (24) Kentucky, Texas, Washington, and other jurisdictions exempt junk foods from tax-free policies applicable to fresh produce, dairy products, and other healthful food choices. In both examples, the brunt of these tax schemes falls hard on products with elevated sodium content. Less clear is whether these taxes are substantial enough to influence consumers away from high salt foods (as has been shown by the substantial tax imposed on sugar-sweetened beverages in Mexico) (25). Spending policies underlying federal and state implementation of food stamp programs encourage recipients to purchase fresh fruits, vegetables, and dairy products but not processed junk foods, which are often loaded with sodium.

Banning sodium in all processed foods is impossible, but partial bans may be viable legally. Limiting or banning access to salty products for minors at school, as discussed above, is lawful just the same as bans on selling tobacco, alcohol, or sugar-sweetened beverages. Prohibiting overuse of sodium as a flavor or preservative in specific products (eg, infant formula) for certain populations (eg, babies) may be legally defensible provided government can demonstrate legitimate, correlated public health concerns.

## Conclusion

Substantially lowering Americans’ sodium consumption requires a multifaceted, public–private strategy that considers legal interventions in tandem with other public health interventions designed to reduce sodium intake through processed and served foods. As with successful voluntary measures, legal steps to curb sodium consumption may have to proceed slowly or in stages to circumvent negative industry or public reactions. Specific legal strategies such as enhanced consumer warnings, litigation, taxation, or partial bans may need to be proven efficacious to sustain their continued or increased use. Some legal interventions may work better than others, at lower cost, and with fewer trade-offs. If the end goal is to reduce Americans’ sodium consumption over time to reduce chronic conditions, efficacious and cost-efficient legal interventions are an essential component of a concerted national plan.
